# Knocking on sports clubs’ doors: A field experiment on ethnic discrimination in Germany

**DOI:** 10.1093/pnasnexus/pgag216

**Published:** 2026-06-18

**Authors:** Cornel Nesseler, Carlos Gomez-Gonzalez, Petr Parshakov, Thadeu Gasparetto, Helmut Dietl

**Affiliations:** Faculty of Social Sciences, University of Stavanger, Stavanger 4021, Norway; Faculty of Social and Political Sciences/Institute of Sports Sciences, University of Lausanne, Lausanne CH-1015, Switzerland; International Laboratory of Intangible-driven Economy, HSE University, Perm 614000, Russia; School of Management SKOLKOVO, Moscow 143025, Russia; College of Applied Human Sciences, West Virginia University, Morgantown, WV 26505, USA; Faculty of Business, Economics and Informatics/Department of Business Administration, University of Zurich, Zurich CH-8032, Switzerland

**Keywords:** discrimination, community organizations, sports, field experiment, intergroup contact

## Abstract

Sports participation gaps persist among certain demographic groups in many countries. Lower participation levels not only compromise the health of these groups but also undermine social cohesion. We conduct a large-scale field experiment to uncover the dynamics of discrimination against immigrants and ethnic minorities across multiple amateur sports. By emailing more than 8,900 sports clubs across seven popular sports in Germany, we find that individuals with native-sounding names are more likely to receive positive responses, with Syrian- and Turkish-sounding names facing the largest differences. The pattern persists across various sports, with some heterogeneity. Openly asking about club membership fees in the email has no influence. The results are robust to alternative models and specifications, including a data-driven causal random forest approach. We discuss implications for governing bodies, attitudes toward foreigners in German society, and future research in this field.

Significance statement Amateur sports clubs are popular community institutions that promote healthy habits and enable intergroup interactions. Yet participation gaps exist and negatively affect ethnic minorities. While self-segregation and preferences may partly explain differences, little is known about discrimination in admission. Using a large-scale correspondence study across thousands of clubs and multiple sports, we find evidence of discrimination against applicants with foreign-sounding names. The magnitude is a 6-percentage-point disadvantage; approximately, a 12% lower probability of receiving a positive response compared with native-sounding names. This discrimination varies across sports but shows no regional patterns. Our findings reveal how individual-level bias aggregates into institutional barriers, undermining the intergroup contact that may enhance social cohesion. The results highlight the need for targeted antidiscrimination interventions.

## Introduction

Amateur sports clubs play an important role in today's society. They promote physical activity, an essential foundation for well-being (1, 2), and foster social integration (3). Participating in amateur sports clubs not only results in a healthy lifestyle but also enhances social cohesion by promoting community engagement (4–6). From a social perspective, amateur sports clubs are social hubs: they bring individuals from diverse backgrounds together and create social bonds that transcend socioeconomic and cultural divides (5–11). The social capital generated through amateur sports improves social integration and creates a sense of belonging (12–14). However, the integrative potential of sports clubs is not unconditional. Research shows that club structures can also reflect and reproduce existing social inequalities, with membership patterns often mirroring ethnic, socioeconomic, and cultural divides (15–17). A fundamental question is the extent to which institutions designed to promote community cohesion, such as sports clubs or cultural associations, may simultaneously reinforce social barriers (18–20).

Participation gaps are persistent between native and foreign-origin individuals in sports (21, 22). Contributing factors may include time constraints (23), financial costs (24), and sports infrastructure (25), yet a long-standing research focus is whether such differences also reflect self-limiting behavior shaped by cultural background (26, 27) and ingroup preferences (6, 28). Such preferences can be reinforced, for example, through intergenerational transmission of norms and attitudes (29), spatial segregation (30), or perceived discrimination (31).

Discrimination is a significant barrier to sports participation for immigrants and ethnic minorities, and a persistent problem in society. Correspondence studies show evidence of discrimination across different societal areas (see e.g. 32 for a review). Different forms of discrimination have been documented in professional sports (see e.g. 33–35), but discrimination is especially problematic in voluntary sports organizations, as it undermines their role as social arenas that foster community engagement and social cohesion (5, 9). Previous research has documented discrimination in amateur football, as individuals with foreign-sounding names are less likely to receive a response when contacting football clubs (18–20). However, the extent of ethnic discrimination in amateur sports, other than football, remains largely undocumented. This gap is particularly important because amateur sports clubs represent one of the most widespread community organizations, serving as primary venues for social interaction across demographic groups. In Germany alone, there are ∼27 million sports club memberships registered (36).

Our study aims to fill this gap by examining ethnic discrimination across multiple amateur sports clubs in Germany using a correspondence study. Specifically, the study examines how amateur sports clubs respond to identical inquiries from potential participants with native- and foreign-sounding names asking to join the club. As in previous studies in football (18–20), we expect individuals with foreign-sounding names to be less likely to receive a positive response. This research sheds light on the barriers that individuals with foreign backgrounds may face in other sports, equally important for health and integration purposes. Our foreign-sounding names represent the three largest foreign population groups in Germany: Turkish, Syrian, and Ukrainian, and we select highly frequent first and last names from these groups in Germany. Choosing foreign names based on frequency aims to ensure that names signal belonging to the respective foreign group and alleviate concerns of systematic associations with other characteristics such as skin tone, religiosity, language proficiency, or socioeconomic status (37).^a^

The sample of clubs includes various team and individual sports, specifically 8,900 German amateur adult clubs for seven of the 10 most popular sports in Germany: football, golf, handball, horse riding, shooting, table tennis, and tennis. The admission process of amateur clubs in Germany is homogeneous, as they operate as voluntary, nonprofit organizations, with low membership fees and the independent capacity to decide whether to admit anyone who applies to be a member (38).^b^ Sports clubs, however, differ significantly in their coverage by popular media, the equipment needed for practice, or the level of community engagement. For example, basic equipment is relatively inexpensive for sports such as football and handball but can be a significant financial burden in sports such as horse riding and golf. Football and, to a lesser extent, tennis and handball, are constantly covered by popular media. Golf, table tennis, and horse riding, however, receive only sparse media attention. Additionally, while shooting clubs are popular in Germany, they work differently from most other popular sports, as socializing and community engagement are often critical.

While our research design is not well suited to shed light on these differences across sports settings, we include a treatment condition to examine the impact of asking about membership fees on the response. By including a treatment that explicitly addresses club membership fees, this study aims to illuminate the interplay between financial and ethnic barriers in sports participation. Membership fees are a well-known challenge common to all amateur sports clubs (39, 40) and a barrier for individuals from lower socioeconomic backgrounds and disadvantaged groups who may not be able to afford them (38, 41). We expect that asking about membership fees will negatively influence the share of positive responses received by applicants with a foreign background. However, it is unclear whether financial barriers disproportionately affect individuals with foreign-sounding names or how clubs perceive these barriers and how they interact with other factors to exacerbate inequalities in sports participation.

## Results

### Descriptive

The number of total responses is as follows. No response: 3,520 (40%); negative response: 256 (3%); positive response: 2,342 (26%); positive response with additional inquiries: 933 (11%); other responses: 840 (9%); and error responses: 1,027 (11%). We create a binary variable equal to 1 for positive responses and 0 for no or negative responses. Following the preregistered strategy, we omit from the analysis “error responses,” and responses marked as “other responses” as they do not directly show clubs’ willingness to invite applicants. The final estimation sample contains 7,051 observations. As predicted, foreign-sounding names receive statistically fewer responses (0.512 vs. 0.449; average treatment effect [ATE] 0.062; *z* = −4.55, *P* = 0.00, *n* = 7,051). This corresponds to a 6-percentage-point difference, or approximately a 12% lower likelihood of receiving a positive response relative to native-sounding names. Additionally, women receive fewer responses, but this difference is not statistically significant (0.455 vs. 0.472; ATE −0.016; *z* = 1.537, *P* = 0.17, *n* = 7,051). Asking about membership fees has no significant influence on the response rate (0.458 vs. 0.472; ATE 0.014; *z* = 1.206, *P* = 0.23, *n* = 7,051). We find, however, heterogeneous results by sport.

Tables 1 and 2 show an overview of the results by sport. Table 1 shows differences between foreign and native-sounding names, which are significant in horse riding, shooting, table tennis, and tennis. People with a foreign-sounding name receive statistically significantly fewer responses. Table 2 illustrates the differences when individuals inquire about membership fees. We find a significant positive effect in football, but a negative effect in shooting and table tennis.

**Table 1:** Response rate by sport: differences between native- and foreign-sounding names.

Sport	Response rate		Difference	*Z*-value	*P*-value	*n*
	German-sounding names	Foreign-sounding names				
Football	0.399	0.414	−0.015	0.454	0.650	1,188
Golf	0.774	0.794	−0.019	0.463	0.643	506
Handball	0.447	0.419	0.029	−0.694	0.488	787
Horse riding	0.281	0.191	0.090	−3.253	0.001	1,204
Shooting	0.511	0.430	0.080	−1.927	0.054	755
Table tennis	0.611	0.497	0.114	−3.627	0.000	1,357
Tennis	0.647	0.576	0.071	−2.213	0.027	1,254

**Table 2:** Response rate by sport: response rate by sport for treatment and control groups.

Sport	Response rate		Difference	*Z*-value	*P*-value	*n*
	Control (baseline message)	Treatment (membership fee question)				
Football	0.383	0.436	−0.053	−1.872	0.061	1,188
Golf	0.804	0.774	0.030	0.817	0.414	506
Handball	0.417	0.434	−0.017	−0.480	0.631	787
Horse riding	0.230	0.197	0.033	1.394	0.163	1,204
Shooting	0.503	0.410	0.093	2.550	0.011	755
Table tennis	0.550	0.501	0.049	1.793	0.073	1,357
Tennis	0.586	0.602	−0.015	−0.546	0.585	1,254

### Main results

**Figure 1:**
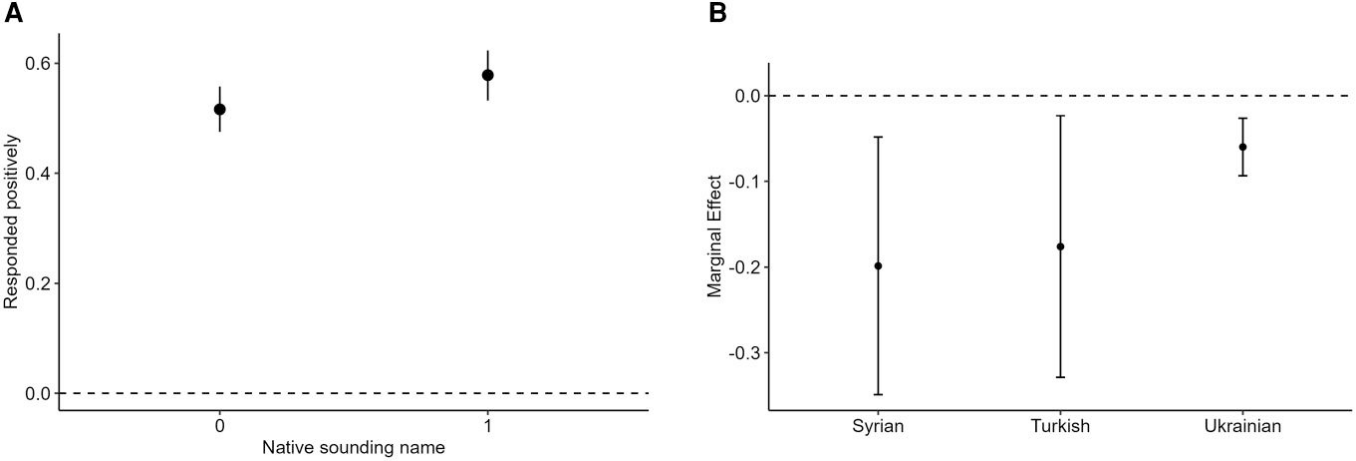
Predictions of probability of response and marginal effects by group (based on the logit model). A) Marginal effect of response for native-sounding names. B) Marginal effect by foreign groups.

Table 3 shows the logit regression results, progressively incorporating a set of control variables in four models. In model 1, we estimate the baseline specification, examining the effect of an email signed with a native-sounding name while controlling for applicant gender, whether the email includes a membership-cost question, whether the club is located in a nonlarge city, the share of foreign residents, and day, sport, and state fixed effects. In Models 2–4, we add further name-related controls. Across all models, the coefficient for a native-sounding name remains statistically significant, underscoring the robust negative influence a foreign-sounding name has on the response rate. The coefficient for native-sounding names is consistently around 0.20–0.25 across all models, with an average marginal effect of 0.06. This means that applicants with native-sounding names are 6 percentage points more likely to receive a response from sports clubs than those with foreign-sounding names. Figure 1A graphically illustrates this finding.

**Table 3:** Logit regression results.

	(1)	(2)	(3)	(4)
(Intercept)	0.02	0.28	1.20*	0.93
	(0.13)	(0.55)	(0.63)	(0.70)
Email is signed with a native-sounding name	0.25***	0.23***		0.18**
	(0.06)	(0.07)		(0.08)
Email is signed with a female-sounding name	−0.15***	−0.14***	−0.12**	−0.13**
	(0.05)	(0.05)	(0.05)	(0.06)
Email includes membership costs question	−0.06	−0.06	−0.06	−0.06
	(0.05)	(0.05)	(0.05)	(0.05)
Club is located in a nonlarge city	0.05	0.05	0.05	0.05
	(0.11)	(0.11)	(0.11)	(0.11)
Share of foreign residents	0.10	0.11	0.11	0.11
	(0.43)	(0.43)	(0.43)	(0.43)
Likelihood that the name is associated with being Christian		−0.34	−1.31	−0.84
		(0.75)	(0.87)	(0.82)
Likelihood that the name is associated with being Muslim		−0.31	−0.38	−0.35
		(0.61)	(0.62)	(0.61)
Email is signed with a Ukrainian-sounding name			−0.27***	
			(0.08)	
Email is signed with a Syrian-sounding name			−0.89***	
			(0.34)	
Email is signed with a Turkish-sounding name			−0.79**	
			(0.35)	
Skin darkness associated with name				−0.21
				(0.14)
Day controls	Yes	Yes	Yes	Yes
Sports controls	Yes	Yes	Yes	Yes
State controls	Yes	Yes	Yes	Yes
Number of observations	7,051	7,051	7,051	7,051
Log-likelihood	−4,482.36	−4,482.17	−4,479.38	−4,481.08
*F*	24.012	22.367	21.060	21.679

Robust SEs are in parentheses. *P < 0.1, **P < 0.05, ***P < 0.01.

In model 2, we add additional control variables regarding the religious associations of names (whether names were perceived as Christian or Muslim). The inclusion of these variables does not alter the significance or magnitude of the native-sounding name effect. The religious association variables are not statistically significant, indicating that the applicant's perceived religion does not significantly influence response rates. In model 3, we disaggregate the foreign-sounding names into Ukrainian-, Syrian-, and Turkish-sounding names. The results show that discrimination is not uniform across these groups. While all foreign groups receive fewer positive responses than native-sounding names, the disadvantage is smaller for Ukrainian-sounding names compared with Syrian- and Turkish-sounding names, as illustrated in Fig. 1B.

In model 4, we add the variable capturing how dark the name is perceived. This variable does not reach statistical significance, indicating that perceptions of skin darkness do not measurably affect the likelihood of receiving a response. The effect of having a native-sounding name, however, remains robust, demonstrating consistent evidence of ethnic bias in responses from sports clubs.

Table 4 shows logit regression models incorporating interaction terms to test whether the native-sounding name effect varies across different groups. Across all models, the native-sounding name remains statistically significant, consistently showing that applicants with native-sounding names are more likely to receive positive responses. None of the tested interactions—(i) email signed with a native-sounding name × having a female name; (ii) email signed with a native-sounding name × asking a membership costs question; and (iii) email signed with a native-sounding name × contacting a club in eastern Germany—are statistically significant in any of the models. This suggests the native-sounding name effect does not differ based on the applicant's gender, whether they asked about membership costs, or whether they applied to clubs in Eastern Germany.

**Table 4:** Logit regression results with set of interactions.

	(1)	(2)	(3)	(4)	(5)
(Intercept)	0.32	0.30	0.29	0.28	0.26
	(0.55)	(0.55)	(0.55)	(0.55)	(0.55)
Email is signed with a native-sounding name	0.20**	0.20**	0.22***	0.23***	0.30**
	(0.09)	(0.10)	(0.08)	(0.08)	(0.15)
Email is signed with a female-sounding name	−0.16***	−0.14***	−0.14***	−0.14***	−0.14***
	(0.06)	(0.05)	(0.05)	(0.05)	(0.05)
Email includes membership costs question	−0.06	−0.08	−0.06	−0.06	−0.06
	(0.05)	(0.06)	(0.05)	(0.05)	(0.05)
Club is located in a nonlarge city	0.05	0.05	0.05	0.04	0.05
	(0.11)	(0.11)	(0.11)	(0.13)	(0.11)
Share of foreign residents	0.10	0.11	0.10	0.11	0.22
	(0.43)	(0.43)	(0.43)	(0.43)	(0.47)
Likelihood that the name is associated with being Christian	−0.38	−0.35	−0.34	−0.34	−0.33
	(0.75)	(0.75)	(0.75)	(0.75)	(0.75)
Likelihood that the name is associated with being Muslim	−0.34	−0.32	−0.31	−0.31	−0.30
	(0.61)	(0.61)	(0.61)	(0.61)	(0.61)
Club is located in eastern Germany			−0.60***	−0.56***	−0.56***
			(0.20)	(0.19)	(0.19)
Email is signed with a native-sounding female name	0.08				
	(0.12)				
Email is signed with a native-sounding name and includes membership costs question		0.07			
		(0.12)			
Email is signed with a native-sounding name and contacts club in eastern Germany			0.14		
			(0.19)		
Email is signed with a native-sounding name and club is located in a nonlarge city				0.04	
				(0.25)	
Email is signed with a native-sounding name and share of foreign residents					−0.45
					(0.85)
Day controls	Yes	Yes	Yes	Yes	Yes
Sports controls	Yes	Yes	Yes	Yes	Yes
State controls	Yes	Yes	Yes	Yes	Yes
Number of observations	7,051	7,051	7,051	7,051	7,051
Log-likelihood	−4,481.95	−4,481.97	−4,481.90	−4,482.16	−4,482.03
*F*	21.627	21.632	21.633	21.632	21.629

Robust standard errors are in parentheses. *P < 0.1, **P < 0.05, ***P < 0.01.

We also run regressions with interactions of native-sounding names with sport and state dummies. While individual coefficients are not reported in the main text, joint tests are informative. The linear hypothesis test for the interaction between native-sounding names and different sports yields a statistically significant result (*χ*^2^ = 13.674, *df* = 6, *P* = 0.0335), indicating that the effect of a native-sounding name varies across sports. In contrast, the joint test for the interaction between native-sounding names and federal states does not produce a statistically significant result (*χ*^2^ = 9.672, df = 15, *P* = 0.8398), indicating consistent effects across German states without regional variation.

Across all models in Tables 3 and 4, we find a significant gender difference, as women are less likely to receive a positive response. The interaction terms, however, show that the ethnic differences are not driven by the applicant's gender. While gender differences were not the focus of this paper and were not preregistered, we provide further insights in the robustness section.

## Discussion

We conduct a large-scale correspondence study by contacting over 8,900 amateur adult clubs in seven popular sports in Germany. Emails are signed with a typical German- or foreign-sounding name. We include a treatment asking how much it costs to join a club. Across various regression models and robustness checks, we find evidence that applicants with native-sounding names enjoy a statistically significant advantage (∼6 percentage points). The findings are consistent with previous research exploring discrimination in various social areas (32, 42), and robust to geographical factors such as club location or the religion/skin color associated with the names used in the study (43, 44).

Discrimination among foreign-sounding groups varies. Syrian- and Turkish-sounding names receive significantly fewer responses than Ukrainian-sounding names. While emails with Ukrainian-sounding names receive fewer responses than those with German-sounding names, the response rate is higher than for the other two foreign groups. The composition of the foreign groups may also explain small differences with previous research that used Italian- and Polish-sounding names in the German football context (20). Similarly, research in Poland has found that individuals with Ukrainian-sounding names receive more favorable treatment than other foreign groups (45). The current political situation and the comparatively more welcoming attitude toward Ukrainian refugees in Germany could partly explain the finding (46, 47). These results suggest that public perceptions of foreign groups may influence how they are treated, opening an interesting avenue for future research. Note that while our focal dimension is the foreignness of names, and we selected highly frequent names and controlled for associations with perceived religion or skin color, we do not formally test for other relevant name associations, such as socioeconomic status (37).

Discrimination also varies across sports. While we find no evidence of discrimination in football or golf, applicants with foreign-sounding names are ∼10 percentage points less likely to receive a positive response in table tennis or horse riding. This pattern suggests that discrimination exists but is not uniform across amateur sports. However, our design does not allow us to identify the mechanisms driving these differences. Simple explanations based on sport-level characteristics, such as team vs. individual sports or the cost of equipment needed to play, appear insufficient. For example, we find no discrimination in golf, a comparatively high-cost sport, while the largest effect occurs in horse riding. Similarly, shooting, often associated with traditional German leisure culture, is not among the most discriminatory sports. These patterns indicate that straightforward categorizations, such as team vs. individual sports, may also provide limited explanatory power. The high overall response rate confirms that correspondence studies are valid across diverse sports settings, laying the groundwork for future research to design sport-specific treatments and isolate potential mechanisms. For example, future research could consider adding treatment arms to compare sports that involve substantial social interaction with those that do not.

Our treatment involving inquiries about membership costs does not significantly affect response rates. Previous research suggests that membership fees and participation costs can represent barriers to joining sports clubs (41) and that financial support measures such as vouchers often have limited effects on participation (38). In our study, however, explicitly asking about membership costs does not reduce the likelihood of receiving a positive response from clubs. Although fee information is sometimes publicly available, we tested whether inquiring about membership fees might signal socioeconomic concerns to club representatives. The results do not support a consistent interpretation, as the effect is negative in some sports and positive in others. As experienced players may already be aware of the fee costs, such inquiries could equally be perceived as a genuine interest in participation. Whether club representatives interpret this question as a signal of limited financial resources or as an indicator of serious intent remains unclear. Future research can help shed light on this issue. Policies aimed at increasing sports club membership should therefore consider how membership fees influence participation alongside other barriers, such as language constraints or transportation costs (48).

Our correspondence study cannot distinguish between statistical discrimination, based on perceived group characteristics, and taste-based discrimination, based on preferences (49). Our names represent three largely represented foreign-origin groups in Germany, which restricts the variation needed to separate these mechanisms. Previous research addressed this by including a larger pool of foreign-origin groups, enabling distinctions based on language proximity and relative sporting performance (19). Replicating this approach across a broad range of sports is less straightforward, as it would require aggregating group-level quality signals across very different sporting contexts. Our findings apply specifically to names linked to Turkish, Syrian, and Ukrainian origins, and discrimination patterns may differ substantially for individuals with names from other foreign backgrounds.

Our findings have broader social implications as individual-level prejudice may aggregate into systematic institutional exclusion. Field experiments show that people readily punish norm violations through both direct confrontation and indirect means (withholding help) but prefer indirect means when possible (50). Our results show that amateur sports clubs may function as sites where such indirect punishment operates, as coaches can easily withhold positive responses from foreign-sounding applicants without direct confrontation, creating exclusion patterns. While humans initially react negatively to increased diversity, adverse effects can be compensated over time through beneficial intergroup contact (51). However, this depends on people actually having access to such contact opportunities. Notably, our results show that discrimination is not uniformly distributed across sports. This pattern means that exclusion may operate selectively, potentially driving foreign-origin individuals away from particular sports over time. Whether actual or perceived discrimination rates create systematic incentives for foreign-origin individuals to avoid certain sports remains an important question that our study cannot directly test (31) and requires future research attention. When community institutions like sports clubs limit access for certain ethnic groups, they prevent the very contact that may build social cohesion over time.

This study has additional limitations that should be considered when interpreting the results. Policy programs often target children. However, our applicants appear to be adults, and the results may not apply to other age groups, such as children or other populations, such as Roma individuals (52). Another issue is that all emails are written in perfect German. This could mean that the average discrimination could be worse than what we find in this study. Moreover, the foreign groups differ substantially in their migration histories. Many Turkish-origin individuals in Germany are second- or third-generation migrants, while Ukrainian and Syrian migrants arrived more recently. These differences in settlement duration may influence how the native population perceives each group in ways this experimental setup cannot capture. Additionally, given data availability and ethical constraints, we do not have information on the respondent's sociodemographic characteristics and cannot assess their association with the response. Future research with access to this information could examine whether an ethnic match between applicant and respondent moderates the likelihood of a positive response, shedding light on the role of ingroup preferences in gatekeeping behavior. Similarly, we lack information on the ethnic composition and diversity of specific clubs and sports, which may shape response patterns and narrow the generalizability of findings to other cultural and voluntary organizations. Finally, we only measure discrimination when people first contact clubs. Other types of discrimination may arise when minorities are already members. Understanding discrimination beyond initial contact and examining experiences within clubs would provide more comprehensive insights. Our experimental setup is scalable, as most European countries share a similar amateur sports system, making it advantageous for future studies.

## Materials and methods

### Experimental setup

Before conducting the experiment, we identified the largest foreign population groups in Germany. While the composition of these groups has changed substantially over the past decade, Turkey remains the largest foreign group in 2024, followed by Ukrainians and Syrians (https://www.destatis.de/). Accordingly, our “foreign-sounding names” category includes names associated with these three origin groups: Turkish, Syrian, and Ukrainian.^c^ We selected highly frequent first and last names from these groups. Additionally, we conducted a small online survey with 50 participants to test whether typical Turkish-, Ukrainian-, and Syrian-sounding names are perceived as foreign by Germans. The survey questions are available in the Supplementary material. The survey participants were also asked to assign a religion and skin color darkness to each name (images from generated photographs showed one face with different skin tones). More than 90% of the respondents correctly assessed the gender of names and whether names sounded foreign. Following the design from similar experiments (18–20), we included these groups. The survey did not ask respondents to associate each name with a specific foreign group. The full list of names and the corresponding survey results are reported in Table S1.

After selecting the names, we created email accounts for each name. We used the first and last names for each email but the same ending for all accounts. All correspondence was conducted in German. We contacted each club once with the following baseline message via email:

Subject: Training session

Hello,

I have recently moved here and would like to join your [name of sport] club. I was in a club before I moved and would like to start again. Is it possible to drop by?

Thank you very much.

Name

Previous research shows that the cost of becoming a member may influence the decision to participate in sports (39, 40). Thus, we include a treatment that examines whether this question influences the supply side by adding one additional sentence in the email before the closing line: “Could you tell me also how much the membership fee is?”

Using block randomization by region, clubs randomly received one email from a native-sounding applicant (*n* = 2,235) or one from one of the three foreign groups (*n* = 6,683). We always used the email address offered by the clubs as the contact information. Some clubs list the email of the coach, assistant coach, or someone working in the administration. However, the user's email address does not always reveal the recipient's position. Additionally, the emails randomly included the baseline email (*n* = 4,217) or the treatment email (*n* = 4,701). Clubs are not necessarily distributed evenly across Germany, and locations differ. In Figs. S1–S7, we include maps showing the distribution of different sports clubs across Germany.

The experiment was conducted from May until July 2024, and emails were sent over a 10-day period (weekdays). In total, we sent out 8,918 emails: football (*n* = 1,513), golf (*n* = 732), handball (*n* = 1,088), horse riding (*n* = 1,501), table tennis (*n* = 1,527), tennis (*n* = 1,529), and shooting (*n* = 1,088). We gathered information about all clubs for the seven sports in Germany. However, following our preregistered power analysis, we limited the number of clubs to ∼1,500 per sport. We randomly selected clubs for the experiment in sports where the initial sample exceeded 1,500 clubs (football, horse riding, table tennis, and tennis) and included the complete population of clubs for the other sports (golf, handball, and shooting).

Most of the responses arrived during the first or second day after the emails were sent out. We stopped checking the accounts after 12 weeks and categorized the responses following a predetermined scheme: (i) no response; (ii) negative response; (iii) positive response; (iv) positive response with additional inquiries (the club/coach asks further questions, i.e. “where did you find about us?” and “which position do you play?”); (v) other responses (the club/coach gives a phone number or another contact means); and (vi) error responses (email not found).

We received ethical approval for the study from the Human Subjects Committee of the Faculty of Economics, Business Administration and Information Technology at the University of Zurich (OEC IRB #2024-045) and preregistered our study, including its primary and secondary outcomes (https://www.socialscienceregistry.org/trials/13430). Participants did not provide prior informed consent; this waiver was approved by the ethics committee, as advance disclosure may incentivize participants to change their behavior or conceal their true preferences, biasing the results. In accordance with the ethics committee guidelines, we did not fully debrief participants; instead, we promptly informed them that the fictitious applicant would no longer be interested in participating. Additionally, we limited contact with participants to a single interaction to minimize any potential burden arising from the deception.

### Econometric model

In the econometric analysis, we quantify the effect of having a native-sounding name on the likelihood of receiving a positive response from sports clubs, using traditional parametric methods. We estimate a series of logit regression models; the general specification is as follows:


$$\log\left(\frac{p}{1-p}\right)=\alpha +\beta_{1}\times \mathrm{nativ}e_{i}+\beta_{2}\times \mathrm{membership}\;+X\gamma +\varepsilon_{i},$$

where *p* is the probability of receiving a positive response, “native” is a dummy indicating that the email was signed with a native-sounding name, “membership” is a binary variable that is equal to 1 if the email includes a question regarding membership costs, and *α* is the intercept. **X** is a vector of control variables including the applicant's gender, an indicator for whether the club is located in a less populated area, the share of foreign residents in the municipality,^d^ sport, and state dummies, and the day the email was sent. As in previous research (18) and following the definition of the German Federal Institute for Research on Building, Urban Affairs and Spatial Development's definition of large cities,^e^ we classify an area as not being part of a large city if the municipality in which the club is located has fewer than 100,000 inhabitants. *ε_i_* is a random error term.

In addition to the main specification, we estimate a series of models that incorporate different sets of control variables and interaction terms. These additional models allow us to examine whether the effect of having a native-sounding name is consistent across various demographic and contextual factors, such as gender, region, and membership cost inquiries. The models also capture the names’ perceived religion and skin tone from the survey. By exploring these interactions, we gain deeper insights into the robustness and potential variations of the observed treatment effect across different subgroups.

### Robustness

We conducted additional analyses using alternative methods and specifications. First, we estimated OLS regression models to confirm that the results remained consistent. The results are available in Table S2. Second, we employed a multinomial logistic regression, which allowed us to treat negative responses and no responses as distinct categories, providing further insight into the nature of club responses. The analysis shows that applicants with foreign-sounding names are not more likely to receive negative responses compared with no responses at all. However, when comparing no response to positive, engaged responses, the native-sounding name variable is statistically significant, with a coefficient of 0.19 (*P* < 0.05). This suggests that applicants with native-sounding names are more likely to receive positive engagement. The effects of other variables remain consistent and are available in Table S3. Additionally, we examined whether response latency varies by name origin. Additional analyses using the number of days until a reply was received do not indicate systematic differences in response speed between native- and foreign-sounding names.

Third, we reclassified other responses as positive to capture any form of engagement from the sports clubs. By classifying these as positive, we capture a broader range of responses that indicate interest, even if they do not directly invite the applicant to participate immediately. The results remain robust across all models, and interestingly, the effect size for native-sounding names increased. The results are available in Table S4. Additionally, we also tested for some specific socioeconomic connotations of names. For example, we compared response rates for Maria, Marie, and Sophie (that may carry stronger middle-class connotations) to those for the other native German names with a strong Christian association. The difference is statistically insignificant.

Fourth, we performed a causal random forest (CRF) analysis, a nonparametric, data-driven way to estimate the treatment effects without imposing the linearity assumptions of traditional models. This method flexibly captures complex interactions and identifies how native-sounding name effects may vary across individuals. The average marginal effect estimated by the CRF is 0.059, which aligns closely with the average marginal effects derived from the logit models (Fig. S8). Figure S9 shows that having a native-sounding name consistently yields positive results across all sports, although the magnitude of the effect varies, with football having the lowest median estimated treatment effect and table tennis the highest.

Finally, we analyze gender differences further. Table S5 shows differences between male- and female-sounding names across the seven sports. This exploratory analysis was not preregistered and lies outside the primary scope of this study; however, the findings may inform future research on gender differences in sport participation. Overall, clubs in our sample responded more frequently to emails sent with male-sounding names than female-sounding names in most sports, although the differences vary in magnitude and statistical significance. The largest disparity is observed in shooting. Horse riding is the only sport in which the pattern reverses: female-sounding names received a significantly higher response rate.

## Supplementary Material

pgag216_Supplementary_Data

## Data Availability

The data supporting the findings of this study are publicly available on Harvard Dataverse: https://doi.org/10.7910/DVN/CLJRPF. All personally identifiable individual data have been removed to protect participant privacy and ensure compliance with ethical guidelines.

## Notes

a Note that respondents may not reliably distinguish between phonetically similar name groups (e.g. Russian- and Ukrainian-sounding names), and perceived foreign origin may partly reflect the relative size of foreign population groups in the local context.

b Our correspondence approach shows that email-based contact with amateur sports clubs is viable across a broad range of sports, as evidenced by an overall response rate of ∼50%.

c
https://www.destatis.de/EN/Themes/Society-Environment/Population/Migration-Integration/Tables/foreigner-place-of-birth.html

d
https://www-genesis.destatis.de/datenbank/online/statistic/12211/details

e
https://www.bbsr.bund.de/BBSR/EN/publications/AnalysenKompakt/Issues/ak-2022-02-dl.pdf?__blob=publicationFile&v=2
