# Supplementary material for: Knocking on sports clubs’ doors: A field experiment on ethnic discrimination in Germany
Source: PNAS Nexus. 2026 Jun 18;5(7):pgag216. doi: 10.1093/pnasnexus/pgag216 (PMC13332438; doi:10.1093/pnasnexus/pgag216)
Supplement: pgag216_Supplementary_Data [file pgag216_supplementary_data.pdf]

Supplementary Material

Knocking on sports clubs' doors: A field experiment on ethnic discrimination in Germany

Supplementary Material A

Table S1. Categorization of names

Names	Correctly categorized as (in %)		Skin color darkness	Assigned the following religion (in %):					
	Male/ Female	Foreign/ Native		Atheist/having no religion	Christian	Muslim	Buddhist	Hindu	Jewish
Abdel Hussain	0.98	0.98	3.09 (1.04)	0.09	0.02	0.89	0	0	0
Ahmet Demir	1	0.98	2.72 (1.04)	0.06	0	0.89	0.04	0	0
Alexander Müller	1	0.98	1.17 (1.04)	0.26	0.72	0.02	0	0	0
Ali Şahin	1	0.98	2.78 (1.04)	0.09	0	0.91	0	0	0
Amira Khalil	0.94	0.98	3.28 (1.04)	0.09	0.04	0.77	0.02	0.06	0.02
Ayşe Kaya	0.96	0.96	2.72 (1.04)	0.09	0.06	0.85	0	0	0
Donya Ismat	0.98	0.98	2.94 (1.04)	0.13	0.13	0.66	0.04	0.02	0.02
Emine Demir	0.89	0.96	2.57 (1.04)	0.11	0.09	0.76	0.04	0	0
Fatima Hussain	1	0.98	3.17 (1.04)	0.06	0.02	0.89	0	0.02	0
Fatma Yilmaz	1	0.98	2.72 (1.04)	0.09	0	0.85	0.07	0	0
Grygoriy Kovalenko	0.96	0.98	1.36 (1.04)	0.19	0.72	0	0.04	0.04	0
Hassan Ismat	1	0.98	3.06 (1.04)	0.09	0	0.87	0.04	0	0
Ibrahim Khalil	1	0.98	3.23 (1.04)	0.11	0.02	0.85	0	0.02	0
Iryna Kovalenko	1	0.96	1.51 (1.04)	0.20	0.67	0.07	0	0.02	0.04
Ivan Melnyk	0.94	0.96	1.65 (1.04)	0.21	0.60	0.09	0.06	0.04	0
Leon Fischer	1	1	1.17 (1.04)	0.28	0.70	0.02	0	0	0
Maria Schneider	1	1	1.21 (1.04)	0.28	0.70	0.02	0	0	0
Marie Müller	1	1	1.17 (1.04)	0.30	0.70	0	0	0	0
Mustafa Kaya	1	0.98	2.70 (1.04)	0.09	0	0.87	0.04	0	0
Oleg Bondarenko	0.94	0.98	1.53 (1.04)	0.23	0.66	0.02	0.06	0.02	0
Paul Schneider	1	0.98	1.11 (1.04)	0.32	0.68	0	0	0	0
Sofiya Melnyk	1	0.94	1.85 (1.04)	0.11	0.49	0.34	0	0.02	0.04
Sophie Schmidt	1	0.98	1.15 (1.04)	0.28	0.72	0	0	0	0
Tetyana Shevchenko	1	0.98	1.62 (1.04)	0.11	0.81	0.09	0	0	0

Table S2. OLS regression results

	1	2	3
(Intercept)	0.50***	0.56***	0.71***
	(0.02)	(0.12)	(0.16)
Email is signed with a native-sounding name	0.06***	0.05***	0.04**
	(0.01)	(0.02)	(0.02)
Email is signed with a female-sounding name	-0.03***	-0.03***	-0.03**
	(0.01)	(0.01)	(0.01)
Email includes membership costs question	-0.01	-0.01	-0.01
	(0.01)	(0.01)	(0.01)
Likelihood that the name is associated to be Christian		-0.08	-0.19
		(0.17)	(0.18)
Likelihood that the name is associated to be Muslim		-0.07	-0.08
		(0.14)	(0.14)
Skin darkness associated with name			-0.05
			(0.03)
Day controls	Yes	Yes	Yes
Sports controls	Yes	Yes	Yes
State controls	Yes	Yes	Yes
Num.Obs.	7051	7051	7051
R^2	0.104	0.104	0.104
Adjusted R^2	0.101	0.100	0.100
Log.Lik.	-4713.635	-4713.434	-4712.310
F	32.520	30.119	29.129

Notes: p < 0.1, ** p < 0.05, *** p < 0.01

Table S3. Multinomial logistic regression results

	Dependent variable:	
	Negative Response (1)	Engaged Response (2)
Email is signed with a native-sounding name	0.22	0.19**
	(0.23)	(0.09)
Email is signed with a female-sounding name	-0.18	-0.14**
	(0.14)	(0.06)
Email includes membership costs question	-0.01	-0.06
	(0.13)	(0.05)
Email is signed with a native-sounding female name	3.38	-0.62
	(2.14)	(0.83)
Email is signed with a native-sounding name and includes membership costs question	2.95*	-0.16
	(1.61)	(0.62)
Skin darkness associated with name	0.13	-0.20
	(0.35)	(0.14)
Constant	-5.98***	0.81
	(1.81)	(0.71)
Day controls	Yes	Yes
State controls	Yes	Yes
Sport controls	Yes	Yes
Akaike Inf. Crit.	10,838.80	10,838.80

Notes: *p<0.1; **p<0.05; ***p<0.01

Table S4. Logit regression results with "other responses" treated as positive category

	1	2	3	4
(Intercept)	0.33***	0.31	0.99*	0.63
	(0.10)	(0.51)	(0.59)	(0.66)
Email is signed with a native-sounding name	0.28***	0.26***		0.24***
	(0.05)	(0.07)		(0.08)
Email is signed with a female-sounding name	-0.12**	-0.12**	-0.10**	-0.11**
	(0.05)	(0.05)	(0.05)	(0.05)
Email includes membership costs question	0.01	0.01	0.01	0.01
	(0.05)	(0.05)	(0.05)	(0.05)
Likelihood that the name is associated to be Christian		0.05	-0.54	-0.20
		(0.70)	(0.82)	(0.77)
Likelihood that the name is associated to be Muslim		0.00	-0.04	-0.02
		(0.57)	(0.58)	(0.57)
Email is signed with a Ukrainian-sounding name			-0.28***	
			(0.07)	
Email is signed with a Syrian-sounding name			-0.66**	
			(0.32)	
Email is signed with a Turkish-sounding name			-0.60*	
			(0.33)	
Skin darkness associated with name				-0.10
				(0.13)
Day controls	Yes	Yes	Yes	Yes
Sport controls	Yes	Yes	Yes	Yes
State controls	Yes	Yes	Yes	Yes
Num.Obs.	7891	7891	7891	7891
Log.Lik.	-5096.217	-5096.104	-5094.930	-5095.801
F	25.211	23.351	21.804	22.536

Note: p < 0.1, ** p < 0.05, *** p < 0.01

Table S5. Response rate by sport. Differences between male- and female-sounding names

Sport	Response rate (N emails)		Difference	Z-Value	P-Value	N
	Male-sounding names	Female-sounding names				
Football	0.422 (963)	0.364 (225)	0.057	1.569	0.117	1,188
Golf	0.819 (248)	0.760 (258)	0.059	1.621	0.105	506
Handball	0.434 (488)	0.411 (299)	0.023	0.635	0.525	787
Horse riding	0.177 (604)	0.248 (600)	-0.071	-3.018	0.003	1,204
Shooting	0.534 (358)	0.375 (397)	0.158	4.363	0.000	755
Table tennis	0.536 (677)	0.513 (680)	0.023	0.847	0.397	1,357
Tennis	0.618 (631)	0.570 (623)	0.048	1.739	0.082	1,254

Note: a. Difference is calculated as the response rate for male-sounding names minus the response rate for female-sounding names. b. Z-values and p-values are based on two-sided two-proportion z-tests.

Figure S1. Overview of football clubs in the experiment by foreign group

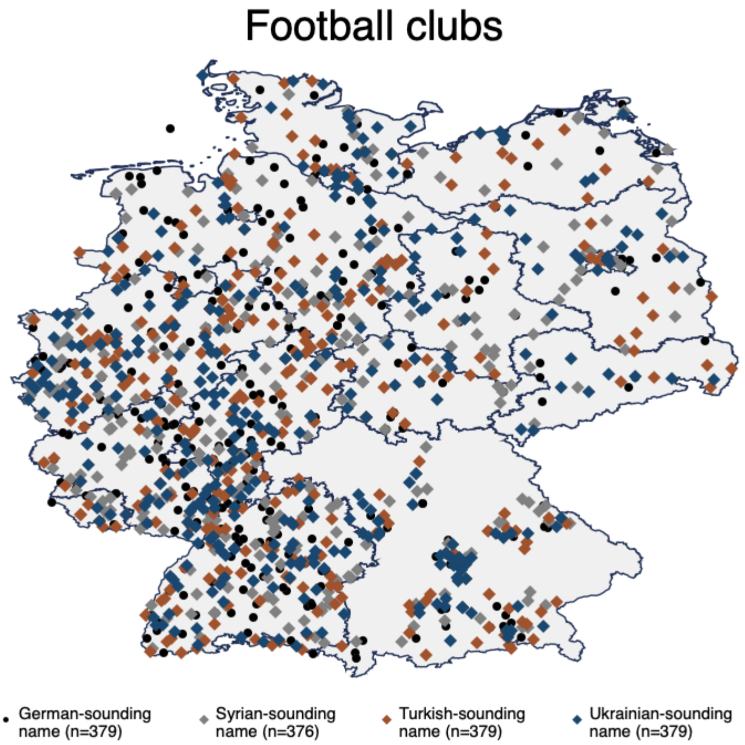

Figure S2. Overview of golf clubs in the experiment by foreign group

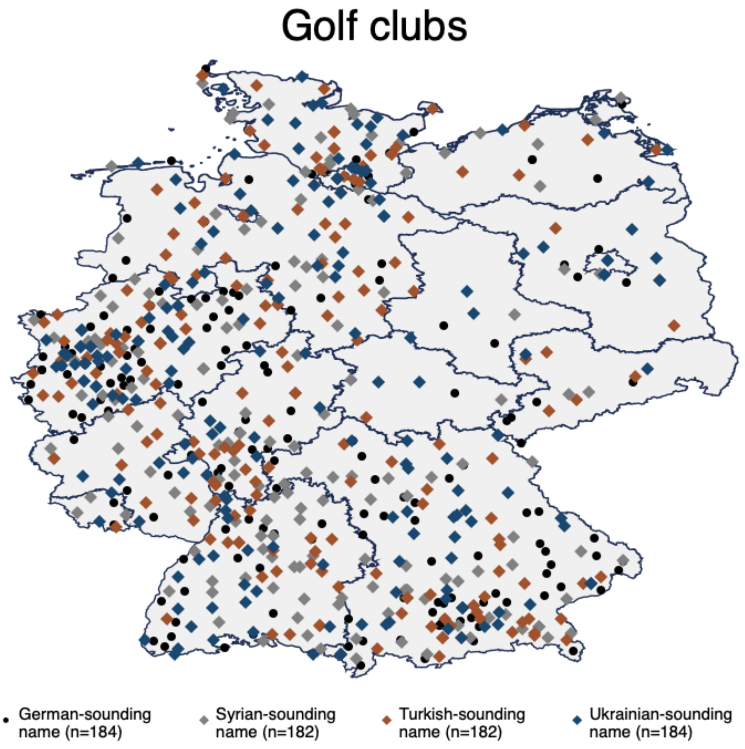

Figure S3. Overview of handball clubs in the experiment by foreign group

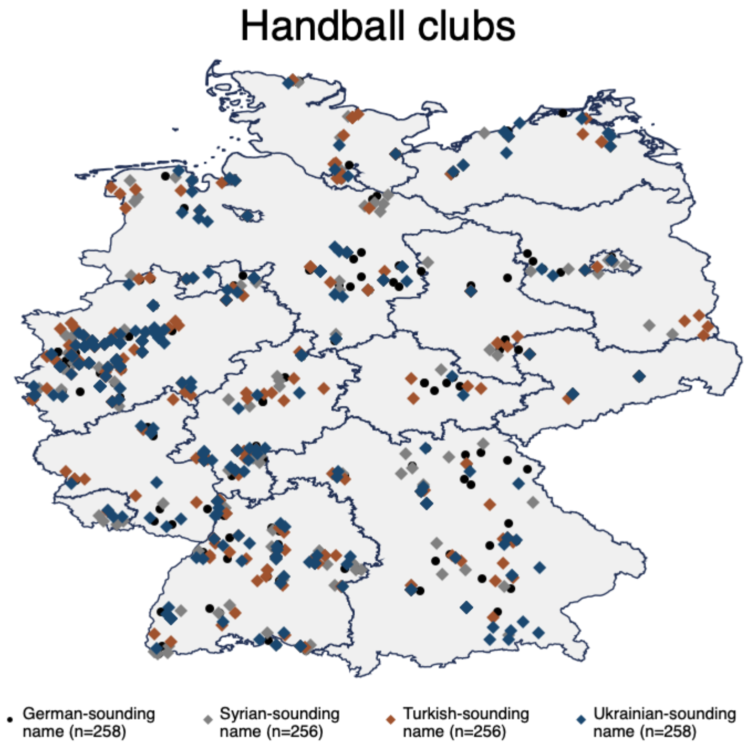

Figure S4. Overview of horse-riding clubs in the experiment by foreign group

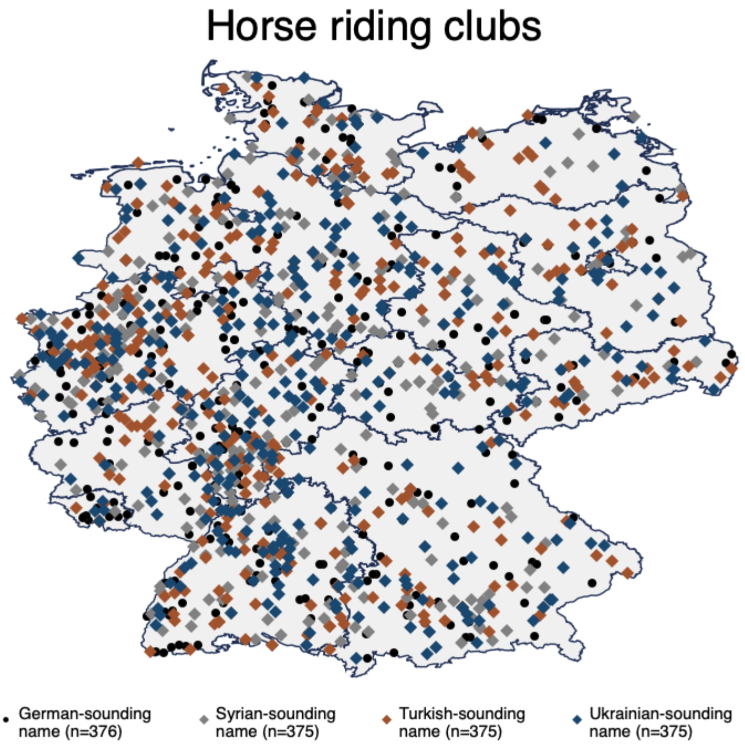

Figure S5. Overview of shooting clubs in the experiment by foreign group

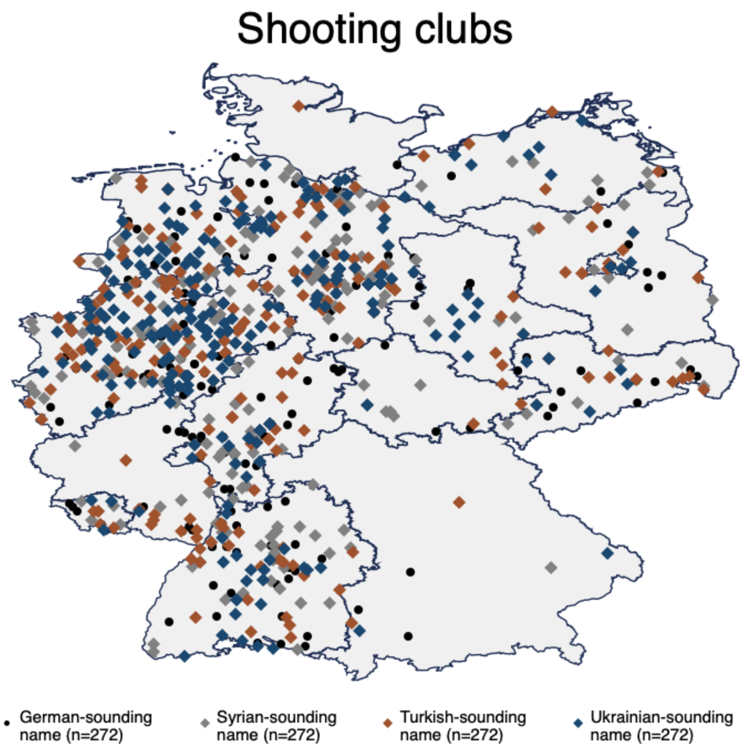

Figure S6. Overview of table tennis clubs in the experiment by foreign group

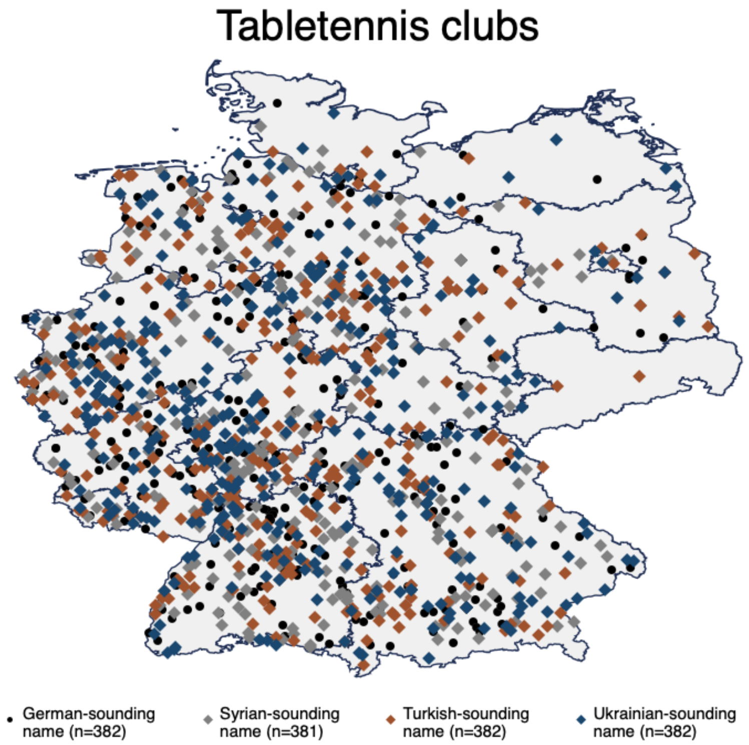

Figure S7. Overview of tennis clubs in the experiment by foreign group

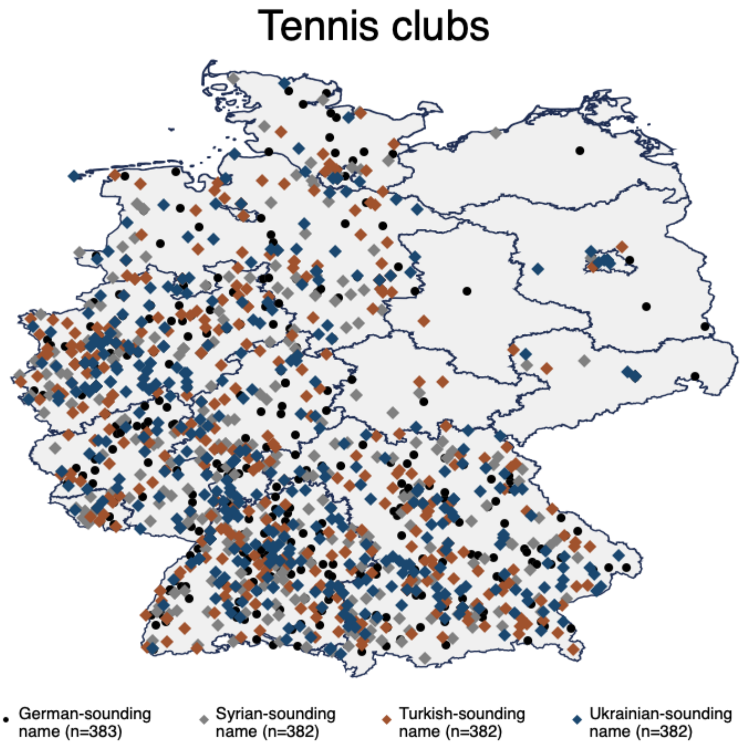

Figure S8. Distribution of marginal effects (Causal Random Forest)

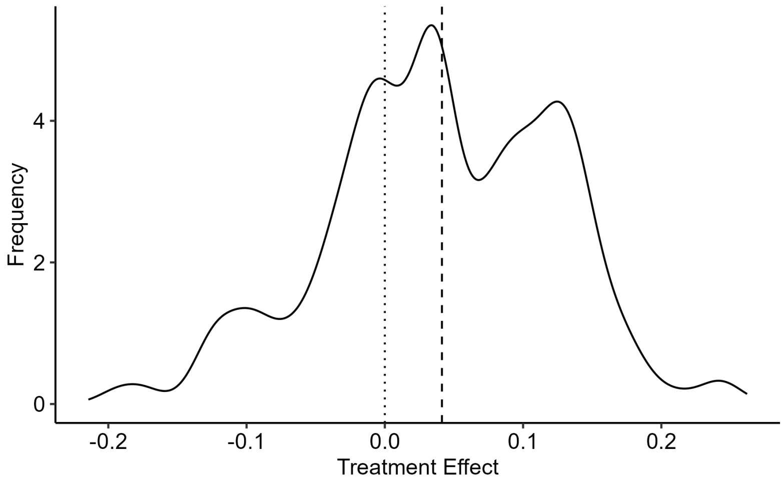

Note: The dotted line is zero, and the dashed line represents the average marginal effect

Figure S9. Marginal effects by sport (Causal Random Forest)

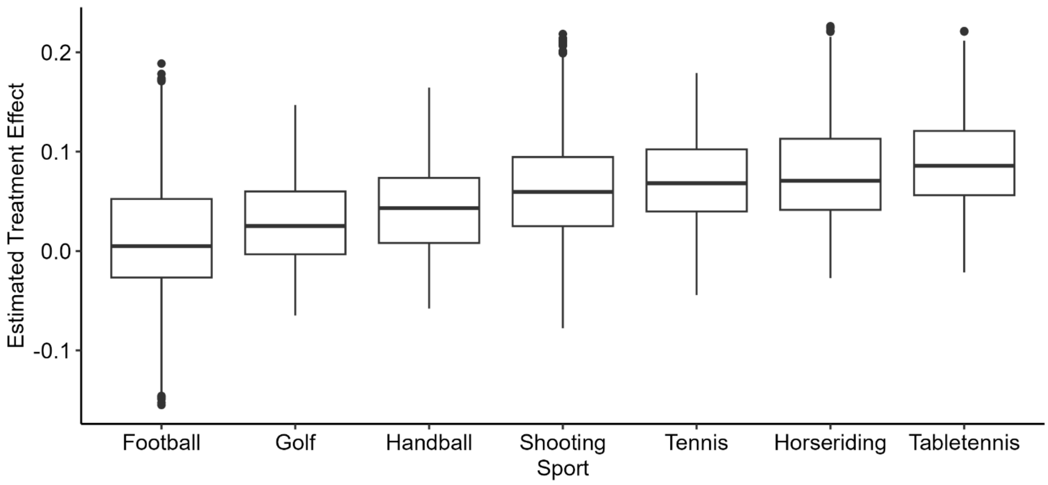

Supplementary Material B

Pre-Experiment Name Survey Questions

Is that a female or male name?

Name	Female	Male
List of names		
…		

What nationality do you associate with (female names)?

Name	German	Syrian	Turkish	Ukrainian	Other
List of names					
…					

What nationality do you associate with (male names)?

Name	German	Turkish	Syrian	Ukrainian	Other
List of names					
…					

Which image do you associate with (female names)?

Name	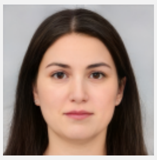	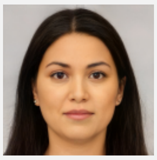	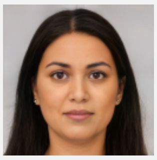	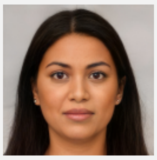	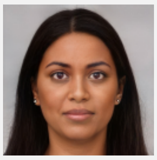
List of names					
…					

Which image do you associate with (male names)?

Name	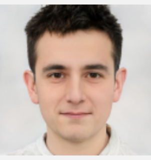	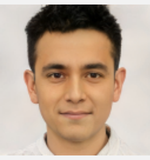	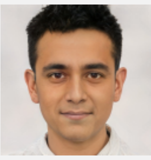	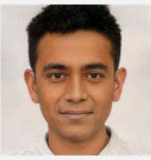	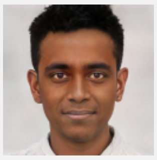
List of names					
…					

What religion do you associate with (female names)?

Name	Atheist / No religion	Christian	Muslim	Buddhist	Hindu	Jewish
List of names						
…						

What religion do you associate with (male names)?

Name	Atheist / No religion	Christian	Muslim	Buddhist	Jewish
List of names					
…					

Attention Check

Every person has a favorite color. To ensure that you are paying attention to the question, please choose the color "White" - independent of what your favorite color might be.

White	Black	Red	Yellow

What is your Prolific ID?
